# Measuring the Effectiveness of Adaptive Random Forest for Handling Concept Drift in Big Data Streams

**DOI:** 10.3390/e23070859

**Published:** 2021-07-04

**Authors:** Abdulaziz O. AlQabbany, Aqil M. Azmi

**Affiliations:** 1 Department of Computer Science, College of Computer & Information Sciences, King Saud University, Riyadh 11543, Saudi Arabia; qabbany@gmail.com; 2 King Abdulaziz City for Science and Technology, Riyadh 12371, Saudi Arabia

**Keywords:** adaptive random forest, data stream, concept drift, online learning, resampling, Poisson distribution

## Abstract

We are living in the age of big data, a majority of which is stream data. The real-time processing of this data requires careful consideration from different perspectives. Concept drift is a change in the data’s underlying distribution, a significant issue, especially when learning from data streams. It requires learners to be adaptive to dynamic changes. Random forest is an ensemble approach that is widely used in classical non-streaming settings of machine learning applications. At the same time, the Adaptive Random Forest (ARF) is a stream learning algorithm that showed promising results in terms of its accuracy and ability to deal with various types of drift. The incoming instances’ continuity allows for their binomial distribution to be approximated to a Poisson(1) distribution. In this study, we propose a mechanism to increase such streaming algorithms’ efficiency by focusing on resampling. Our measure, resampling effectiveness (ρ), fuses the two most essential aspects in online learning; accuracy and execution time. We use six different synthetic data sets, each having a different type of drift, to empirically select the parameter λ of the Poisson distribution that yields the best value for ρ. By comparing the standard ARF with its tuned variations, we show that ARF performance can be enhanced by tackling this important aspect. Finally, we present three case studies from different contexts to test our proposed enhancement method and demonstrate its effectiveness in processing large data sets: (a) Amazon customer reviews (written in English), (b) hotel reviews (in Arabic), and (c) real-time aspect-based sentiment analysis of COVID-19-related tweets in the United States during April 2020. Results indicate that our proposed method of enhancement exhibited considerable improvement in most of the situations.

## 1. Introduction

Big data has become central to our digital landscape, and an enormous volume of data is generated every second by innumerable sources. A number of algorithms and solutions have been proposed to deal with the tremendous amount of data.

Most data mining and machine learning approaches incorrectly assume that examples are i.i.d (independent and identically distributed) and generated from a stationary distribution. Currently, we are bombarded with a massive amount of distributed data that are generated from an ever-growing number of smart devices. In many cases, this data may not even be saved. Our ability to collect data is also changing dramatically. Computers and small devices send data to other devices; thus, we are faced with the presence of distributed sources of data and a continuous flow of data generated from non-stationary processes. Some examples of data mining applications in this context are sensor networks, social networks, web mining, radio frequency identification, and financial data [1].

Predictive analytics is a branch of advanced analytics that is used to predict uncertain future events. It uses many techniques ranging from data mining, statistics, modeling, machine learning, and artificial intelligence (AI) to analyze the current data to make predictions about the future [2,3,4]. Predictive analytics is a main area in big data research owing to the critical need for predictions in several fields. Many studies were conducted on making forecasts based on historical data; however, with the advent of advanced technologies, such as cloud computing and the Internet of Things (IoT), stream learning has garnered more attention. Moreover, the ever-increasing amount of data generated every day makes data streams or online learning all the more important.

Accuracy is fundamental in building reliable prediction applications, and a lack of high accuracy in such systems renders them useless. Therefore, continuous monitoring of prediction applications is crucial for detecting any deviations in their performance. These deviations are called “concept drift,” a term that specifically refers to the behavior of the prediction model when it is affected negatively by sudden changes in the input variables. As a result, there is a corresponding decrease in the accuracy of the generated predictions. Thus, it is important to enhance the ability to handle these variations effectively.

Several reasons may lead to concept drift in daily-life applications, including, but not limited to, intrusion detection, equipment failure, seasonal changes in water temperature or weather, e-mail spam filtering, the detection of sensor data of unusual movement, and many others [5].

Another area that may benefit from concept drift is sentiment analysis, where we classify emotions within text data using textual analysis techniques. In the last decade, many research studies were conducted in the field of sentiment analysis and opinion mining, especially with regard to social networks and customer comments and reviews. However, with the massive and rapid increase in digital data, there is an urgent need for more in-depth research, taking into account the importance of real-time analysis, an analysis that addresses the topic from a more detailed angle such as effective handling of concept drift. This will allow the field to keep pace with the overwhelming increase of user dependency on digital solutions worldwide.

In machine learning, ensemble methods use multiple classifiers whose results are combined. The objective is to obtain a better predictive performance than that obtained from any of the individual classifiers [6]. Two of the most widely known ensemble methods are boosting [7] and bagging [8]; a more recent addition is random forest [9]. Random forest (or random decision forest) is an ensemble learning method for classification and regression that builds multiple decision trees and merges them to obtain a more accurate and stable prediction. It is widely used in the classical non-streaming settings of machine learning applications [9]. Gomes et al. [10] stated that there is no actual algorithm that can be considered to be state-of-the-art for employing the random forest method for stream learning. Thus, they introduced the Adaptive Random Forest (ARF) algorithm to deal with evolving data streams on the fly. We assume that there is room for further enhancement.

In this study, we adopted the ARF algorithm and showed that it is possible to increase its efficiency by concentrating on the resampling method. We summarize our contribution as follows:

- We propose a new measure that determines the effectiveness of the resampling method.
- We apply the measure on synthetic data sets to select the best parameter.
- We use three case studies to validate our choice of the parameter. Two of the studies involve customer reviews, and the third is aspect-based sentiment analysis.

We call the proposed measure “resampling effectiveness” (denoted ρ). The measure considers the speed and the efficiency by which the system adapts to the concept drift. It comprises the accuracy as well as time factor to determine the usefulness of resampling. We used six synthetic data sets to empirically determine the best parameter to yield higher effectiveness in resampling. The synthetic data sets incorporate all the known types of concept drift. Three case studies were used to validate our choice of the parameter. These were as follows: (a) Amazon customer reviews (English data set), (b) hotel reviews from Booking.com covering the period Jun/Jul 2016 (Arabic data set), and (c) real-time aspect-based sentiment analysis of tweets related to COVID-19 in the United States during the month of April 2020.

This paper is organized as follows: Section 2 presents the background of the problem and related work. The proposed measure is outlined in Section 3. In Section 4, we describe the experiments with different settings and discuss the results. The three case studies are detailed in Section 5, Section 6 and Section 7. In Section 8, we present the conclusions and our outlook for future work.

## 2. Background

Concept drift represents unforeseen modifications in the underlying distribution of streaming information over time [11]. Studies on this subject include the advancement of philosophies and procedures for detection of the drift, its understanding, and adaptation. In this section, we consider concept drift and its handling in some detail; in addition, we examine the features of aspect-based sentiment analysis.

### 2.1. Concept Drift

Analyzing data streams is a challenging issue as it requires a continuous examination of the received data. This is because hidden insights and patterns, as well as the data, are expected to evolve; therefore, stream or online learning applications are prone to lose their accuracy if they are not monitored appropriately.

Concept drift describes a phenomenon that occurs when the accuracy of the prediction model starts to decrease over time, which results in the model becoming obsolete after a short while. Therefore, there is a critical need for advanced capabilities that can be leveraged to adapt perfectly to the changes that occur. Just to explain, concept drift is caused by alterations in the underlying distribution of incoming data [12]. These altered data negatively affect the entire model if they are treated as normal. The prediction model must be smart enough to detect any variation in the input data and treat it immediately and effectively. In particular, the model should anticipate the occurrence of concept drift so that the readiness and preparation take place before the moment of its occurrence. This would ensure that there is no interruption or delay when replacing the prediction model.

**Figure 1:**
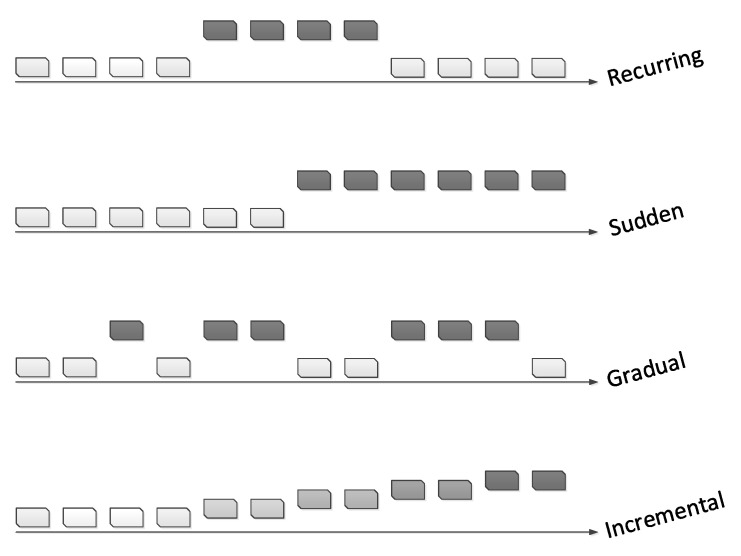
Different types of concept drift. Reproduced from [13].

Generally, there are four types of concept drifts, as shown in Figure 1. The first step in tackling any problem is to understand it well. Lu et al. [11] pointed out that an understanding of concept drift can be accomplished by defining the following factors: occurrence time, period of concept drift (when), degree or severity of drifting cases (how), and the affected area or region of the feature space (where).

Many reasons may lead to concept drift in daily life. We give examples of real-life problems involving concept drift. Zenisek et al. [14] presented a concept drift-based approach for improving industrial processes of predictive maintenance, which helped estimate when the maintenance operations must be performed. They carried out real-time monitoring and analysis of equipped sensor data to detect and handle any drift caused by a malfunction in the process. After conducting a real-world case study on radial fans, the authors discussed the potential benefits gained from such an approach for saving time and material and improving the overall process. Similarly, Xu et al. [15] drew attention to the risks of accumulating digital data in smart cities as a result of the tremendous advances in AI and IoT technologies, which raise the level of information security threats. Therefore, they suggested activating anomaly detection techniques that take into account the concept drift issue that is expected to occur with time on data flow. After conducting their experiment on a real data set, the authors described the effectiveness of their method, which reflects what machine learning can do for cyber security.

Saadallah et al. [16] discussed the handling of concept drift in real-time decision systems of transportation networks. The authors conducted taxi demand prediction experiments in three large cities: Porto (Portugal), Shanghai (China), and Stockholm (Sweden). They demonstrated that the proposed drift-aware framework reflected good results in terms of prediction accuracy and concept drift handling.

Historically, concept drift was handled using different philosophies. We highlight some well-known approaches below.

#### 2.1.1. Mining Data Streams

During the last decade, numerous approaches and algorithms were proposed to deal with evolving data streams [17]. The ensemble model is one of the models that stream learning researchers are increasingly interested in, owing to its efficacy and feasibility.

Ensemble learning algorithms combine the predictions of multiple base models, each of which is learned using a traditional algorithm such as decision tree; thus, a single classifier’s predictive accuracy will be collectively enhanced [18,19]. Many studies have applied the ensemble model to the stream learning approach to deal with issues of concept drift handling [20].

Bagging and boosting are widely-used ensemble learning algorithms that were shown to be very effective compared with individual base models in improving performance. Bagging operates by resampling (random sampling with replacement) the original training set of size *N* to produce several training sets of the same size, each of which is used to train a base model [21,22]. Originally, these learning algorithms were intended for batch/offline training. In [23,24], an online version of the bagging and boosting algorithms was introduced. Online learning algorithms process each training instance on arrival without the need for storing or reprocessing. The system exhibits all the training instances seen so far.

Let *N* be the size of the training data set, then the probability of success is p=1/N, as each of the items is equally likely to be selected. The process of bagging creates a set of *M* base models, and each of them is trained on a bootstrap sample of size *N* through random sampling with replacement. The training set of each base model contains *K* times the original training examples given by the binomial distribution (Equation (1)),

(1) Pr(K=k)=Nkpk(1−p)N−k.

Binomial and Poisson are well-known distributions in probability theory and statistics and were the backbone of several algorithms in machine learning. Coupled together, the binomial distribution can converge to the Poisson as an approximation when the number of trials is large, and the probability of success in any particular trial approaches zero, i.e., p→0. Let λ=Np, then it can be shown that as N→∞ we obtain

(2) $$\mathrm{Pr}\left(K=k\right)=\frac{\lambda^{k}}{k!}e^{-\lambda }.$$

The λ is the expected number of events in the interval and is also known as the rate parameter. Equation (2) is the probability density function for the Poisson distribution, denoted by Poisson(λ). Oza and Russell [23] argued that in online bagging, the incoming streaming data could be considered as an unlimited training data set in which N→∞, and the distribution of *K* tends to a Poisson distribution with λ=1, as given in Equation (3),

(3) $$\mathrm{Pr}\left(K=k\right)=\frac{e^{-1}}{k!}.$$

Here, instead of sampling with replacement, each example is given a weight according to Poisson(1). A wide range of studies [23,24] was carried out in this context to find an optimal solution for handling concept drift. For instance, Bifet and Gavalda [25] used variable size windows for handling more than one type of concept drift simultaneously. They proposed “Adaptive Windowing” (ADWIN), an algorithm that automatically adjusts the window size based on changes detected in the model’s behavior, i.e., a dynamic monitor for detecting drifting cases was incorporated. The results showed that the proposed technique outperformed the fixed sliding window-based approach in terms of adaption behavior. The algorithm proposed in [25] was inspired by the work in [26], a well-known study in this field.

Du et al. [27] developed a sliding window-based method using entropy for adaptive handling of concept drift. It could determine the appropriate timestamp for retraining the model when the drift occurred. The authors stated that the windows were monitored dynamically to assess their entropy. When the drift occurred, the time window was divided into two parts to calculate the average distance between them. The algorithm then detected the most suitable point for rebuilding the model. Regarding the assessment, five artificial and two real data sets were tested in the experiment. The outcomes revealed outstanding results in terms of recall, precision, and mean delay.

Furthermore, Khamassi et al. [28] conducted a study in which they tracked concept drift occurrence using two sliding windows. The first window is a self-adaptive variable window that expands and contracts based on the detected drifts. The second window contained a batch of collected instances between two determined errors. The objective was to apply a statistical hypothesis test for comparing the distribution of error distance between the windows. The results of the study showed an early detection of drifts and a minimized false alarm.

Liu et al. [29] proposed a fuzzy-based windowing adaptation method that allowed sliding windows to maintain overlapping periods. The data instances were weighted by membership grades to differentiate between the old and new concepts. After evaluating their proposed approach by several data collections, the authors stated that their method yielded a more precise determination of the instances that belonged to different concepts, which in turn enhanced the learning model adaption.

Yang and Fong [30] presented an optimized version of the very fast decision tree (VFDT) [31]. Their system prevented the explosion of the tree size and minimized the degradation of accuracy resulting from noise. The incrementally optimized very fast decision tree (iOVFDT) algorithm used a multiple optimization cost function that took into consideration three important aspects, namely accuracy, tree size, and running time. Subsequently, in [32], the same team of researchers conducted a study comparing the performance of their proposed algorithms with VFDT [31] and ADWIN [25] regarding the handling of concept drift. The authors concluded that iOVFDT achieved the best results in terms of accuracy and memory usage.

Krawczyk and Woźniak [33] suggested using a modified weighted one-class SVM to tackle concept drift cases adequately. They clarified specifically that the data stream was processed as consecutive data chunks with the possibility of having concept drift in one of them, and each data chunk would be trained and tested on the following one. They used artificial and real data sets to evaluate their approach, and the outcomes revealed promising results compared to the one-class very fast decision tree (OcVFDT) [34]. Similarly, Pratama et al. [35] proposed an evolving fuzzy system to handle concept drifts in colossal data streams by employing a recurrent version of the type-2 fuzzy neural network. They demonstrated that their approach was flexible enough to be modified to comply with the requirements of the learning problem.

Krawczyk et al. [36] conducted a comprehensive survey regarding ensemble learning for analyzing data streams. They used the data processing mechanism as the main factor for categorizing the ensemble approach of handling concept drift, i.e., they differentiated between data stream instances being processed individually or collectively (by a chunk-based method). In addition, the study explored several research paths under the umbrella of the ensemble model, and work was carried out in stream learning. The authors presented open challenges and potential redirection in this important field, e.g., they discussed the need for high-performance capabilities for big data applications. The study suggested methods to improve the scalability of the proposed algorithms or using dedicated big data analytics environments such as Spark [37] or Hadoop [38]. Of course, the aim was to use these types of environments by taking into account the phenomenon of concept drift and the streaming nature of incoming data; some studies have proposed good approaches using big data technologies as in [39], but in classical (batch) machine learning settings.

#### 2.1.2. Random Forest and Its Adaptive Version

On surveying the literature on concept drift issues, we observed that some well-known machine learning algorithms, such as the random forest algorithm, have not been addressed extensively. Random forest is an ensemble learning model that is widely used in classical non-streaming settings. As stated by Marsland [40], “If there is one method in machine learning that has grown in popularity over the last few years, it is the idea of random forests”. However, the situation is completely different for stream-based learning studies, and few studies have been published concerning the application of the random forest algorithm for handling concept drift.

Abdulsalam et al. [41] presented a streaming version of the random forest algorithm that used node and tree windows. Their approach, however, does not take concept drift into consideration as the data streams were assumed to be stationary. Therefore, Reference [42] discussed dynamic streaming random forests to deal with evolving data streams that were prone to changes over time. Their work was an extension of their previous work that added the use of tree windows of flexible size in addition to an entropy-based concept drift detection technique. Subsequently, Saffari et al. [43] introduced online random forests that combined the ideas of online bagging and extremely randomized forests. They also used a weighting scheme for discarding some trees and starting to grow new ones based on the out-of-bag (OOB) error measure found at certain time intervals. There were some further studies in this field, but no remarkable results were reported regarding the handling of concept drift.

Gomes et al. [10] proposed the Adaptive Random Forest (ARF) algorithm for classifying evolving data streams. Their approach toward evolving data streams employed resampling along with an adaptive strategy that provided drift monitoring for each tree in two steps: the first was direct training of a new tree when a warning was issued, and in the second step, the tree was instantly replaced if concept drift occurred. The authors compared their proposed approach with different state-of-the-art algorithms, and the results demonstrated its accuracy. Another key point is that a parallel version of the algorithm was built that was found to be three times faster than the serial one.

Some streaming algorithms can handle only one type of concept drift; however, ARF can cope with various types. This competitive advantage motivated us to study ARF in more depth to find possible enhancements.

### 2.2. Sentiment Analysis

As we stated earlier, sentiment analysis is an ever-growing field involving the interpretation and classification of emotions within different data mediums using data analytics techniques. Opinion mining has become a complicated task owing to companies and agencies looking for a detailed and real-time analysis of user experiences and aspirations. As a result, recent research studies have attempted to uncover various factors indicating the polarity of the assorted aspects included in the text being analyzed. These days, aspect-based sentiment analysis (AbSA) is a promising research area that is being widely discussed, e.g., [44,45]. Dragoni et al. [46,47] implemented an opinion mining service that extracted polarities referring to specific aspects included in the processed texts. According to them, the detection of these aspects was critical, especially when their sources did not belong to known domains. Thus, the Open Information Extraction (OIE) strategy [48] was utilized as an unsupervised aspect-based analysis approach. The algorithm developed the grammar dependency graph to extract any aspect and opinion referring to them. The outcome of their study reflected the feasibility of the proposed algorithm notwithstanding a certain lack of accuracy and recall that may be attributed to the use of an unsupervised model.

One aspect that requires further investigation in the field of sentiment analysis is a time dimension that would produce a clearer and broader view of the sentiments. Ibrahim and Wang [49] conducted a study on the perception of customers about certain brands of online products. They analyzed a large number of tweets associated with five prominent retailers, during a massive sales period, to explore trends in customer sentiments and impressions. Using advanced techniques, such as time series analysis and topic modeling, they attempted to understand the reasons behind the changes resulting from significant deviations in the sentiments and opinions of people at certain critical time points.

Certainly, the accuracy of sentiment analysis is subject to a decline over time if continuous updating is not overseen. Rubtsova [50] discussed this issue and offered three different mechanisms to improve the classification system of sentiment analysis. The first used a term frequency-inverse corpus frequency formula for updating the model regularly, while the second approach utilized an external emotional or evaluative vocabulary, or both, to enhance the quality of the classification system. The last mechanism tackled the usage of the distributed word representations as a *k*-dimensional feature space. The author stated that all the examined approaches showed good results in reducing the deterioration of the sentiment classification model. Furthermore, another challenge to the accuracy is that the feeling of certain words in a context differs with different aspects; and it is impossible to deduce this from the context words alone. Such an issue was explored by Shuang et al. [51], who proposed a mechanism that consisted of a special layer for distinguishing between the aspect-relevant features of words in a context.

Sentiment analysis provides an opportunity to maximize the benefits of utilizing user opinions and comments proliferating over the Internet. To illustrate this, Bi et al. [52] suggested applying the Asymmetric Impact Performance Analysis (AIPA) technique to online reviews of customers instead of regular surveys. Consequently, they conducted a case study on the reviews of a five-star hotel that were submitted by its guests through Tripadvisor.com (accessed on 4 July 2021),a well-known tourism website. Several features such as transport, cleanliness, service, food, and facilities were assessed to measure the customers’ feelings towards the hotel. The results reflected the feasibility of the proposed approach in terms of low cost and short time associated with customer satisfaction analysis.

## 3. Our Proposed Measure

At its core, the ARF (Adaptive Random Forest) algorithm depends heavily on the aggregating process of online bootstrapping. All new learning instances are likely to be chosen zero or *k* times following a binomial distribution. In addition, with a huge sample size as with stream learning, this distribution can be approximated to a Poisson(1) distribution, as mentioned earlier. For data streams, Bifet et al. [53] proposed a variant of bagging called leveraging bagging. To enhance the process, they argued for using a larger value of λ to compute the value of the Poisson distribution that would increase the weight of resampling, which in turn would improve the performance. For that, they chose λ=6 without a thorough investigation or proper justification. Nevertheless, we need a satisfactory basis for choosing this or any other value of λ for use in the ARF algorithm.

**Figure 2:**
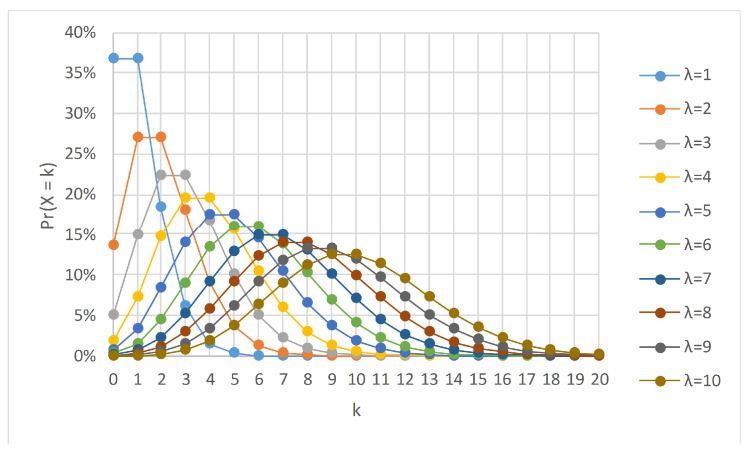
Mass function of Poisson distribution. The function is defined only at integer values of *k*; the connecting lines are visual guides for the eye.

Figure 2 shows several Poisson distributions with different values of λ. It is worth noting that the mean and variance of a Poisson distribution is λ. The area under the curve reflects the behavior of the training model, i.e., it gives an indication of the extent to which a new training example will be used for updating the base model. For example, when λ=1, we see that 37% of the values are 0, 37% are 1, and 26% are values greater than 1. That is, we are taking out 37% and repeating 26% of the examples. The online bagging [23] algorithm used Poisson(1) for simulating the resampling process with replacement, while Bifet et al. [53] used Poisson(6). Gomes et al. [10] used the same value of λ in ARF as in leveraging bagging, i.e., Poisson(6). However, this raises several questions that need to be addressed:

- Is it necessary that the appropriate value of λ for the leveraging bagging be suitable for ARF, in general?
- Does changing the value of λ definitely impact the performance?
- What is the overhead associated with using a larger value of λ?

The present study tries to address these concerns by proposing a measure and using it to assess different values of λ in the ARF algorithm to determine the one that is most suitable.

### Resampling Effectiveness (ρ)

The ARF algorithm in [53] used a higher value of λ, claiming that it increased the likelihood of instances getting higher weights during the training stage of the base model. However, it is necessary to determine the criterion by which this increase is effective. Moreover, the goal of accuracy must not be attained at the expense of processing time and vice versa. Therefore, we introduce a measure that takes into consideration the accuracy as well as processing time to determine the efficacy of the resampling method.

Let $\alpha_{\mathrm{rank}}$ be the normalized accuracy rank, and $\tau_{\mathrm{rank}}$ be the normalized execution time rank. We define these as

(4) $$\begin{array}{cc} \alpha_{\mathrm{rank}} & =10+90\left(\frac{\alpha -\alpha_{\min}}{\alpha_{\max}-\alpha_{\min}}\right), \end{array}$$

(5) $$\begin{array}{cc} \tau_{\mathrm{rank}} & =100-90\left(\frac{\tau -\tau_{\min}}{\tau_{\max}-\tau_{\min}}\right), \end{array}$$

where α is the current accuracy obtained at a specific value of λ, and $\alpha_{\min}$ and $\alpha_{\max}$ are the minimum and maximum accuracies achieved, respectively, for all the values of λ in the range

(6) $$\begin{array}{cc} \alpha_{\min} & =\min_{\lambda }\;\left\{\alpha \right\}, \end{array}$$

(7) $$\begin{array}{cc} \alpha_{\max} & =\max_{\lambda }\;\left\{\alpha \right\}. \end{array}$$

We call it normalized rank since we assign a value of 10 for the worst accuracy and a value of 100 for the best. This normalization allows for proper handling of accuracy, no matter how narrow or wide the difference between its maximum and minimum values. Similarly, let τ, $\tau_{\min}$ and $\tau_{\max}$ be the actual, minimum, and maximum execution times, respectively, at a specific value of λ. The $\tau_{\mathrm{rank}}$ is a normalized reverse rank. As we prefer a shorter execution time, we assign a value of 10 to the longest execution time and 100 to the shortest. We combine both normalized ranks into a single measure. The resampling effectiveness, which we denote ρ, is the harmonic mean of both ranks and is given by,

(8) $$\begin{array}{cc} \rho & =\frac{2}{1/\alpha_{\mathrm{rank}}+1/\tau_{\mathrm{rank}}} \end{array}$$

(9) $$\begin{array}{cc} \; & =\frac{2\;\alpha_{\mathrm{rank}}\;\tau_{\mathrm{rank}}}{\alpha_{\mathrm{rank}}+\tau_{\mathrm{rank}}}. \end{array}$$

The value of ρ ranges from 10 to 100, with a higher value signifying a more effective resampling.

## 4. Picking the Best Value of λ

In this section, we describe the conducted experiments to empirically determine the best value of λ to be used with the ARF algorithm. We examined the value of λ∈[1,10] because an excellent Poisson approximation can be reached if λ≤10 [54]. The best value of λ corresponds to the one that results in the highest value of ρ.

The nature of concept drift calls for further research experiments within an empirical research framework that uses evidence-based data. For example, Santos et al. [55] proposed an empirical method that tackles differential evolution for guiding the tuning process of concept drift detectors in order to enhance their accuracies. Moreover, there are many researches in other computing areas that employ empirically derived settings. For instance, Google’s PageRank uses a damping factor of 0.85 [56], since by practice it was found that a good choice would be between 0.8 and 0.9, though in theory, it could be any value between 0 and 1.

We used six different data sets, with each having a different type of drift. Each experiment was repeated multiple times to avoid any inconsistency in the results.

### 4.1. Data Sets

For this experiment we used six different synthetic data sets, which are publicly available, to diversify the resources. There are several benefits of using synthetic data: they are easy to reproduce and cost little to store and transmit. Synthetic data provide an advantage of knowing the ground truth (e.g., where exactly the concept drift occurs) [57]. We briefly describe these data sets below:

- **LED** (LED generator). This data set was introduced by Breiman et al. [58] for predicting the digits appearing on a seven-segmented LED display. This data set contains 7 Boolean attributes (after the seven light-emitting diodes in the LED display) and 10 concepts (the set of decimal digits). The authors introduced noise into the data set, thus the value of each attribute had a 10% chance of being inverted. The optimal Bayes classification accuracy was 74%.
- **SEA**. This data set was used by Street and Kim [59] for testing their proposed streaming ensemble algorithm (SEA). The data contain abrupt concept drifts. It has three independent real-valued attributes (data from the United States Census Bureau, breast cancer data set from SEER program, and anonymous web browsing) in the range of 0–10, two of which are relevant for prediction.
- **AGR** (AGRAWAL). This data set is based on the generator devised by Agrawal et al. [60]. They produced a data stream with six nominal and three continuous attributes. The processed instances are mapped into two distinct classes. A perturbation factor is used to add noise to the data.
- **RTG** (RTG generator). This random tree generator was part of the work by Domingos and Hulten [31] in their distinctive algorithm VFDT. They fabricated a decision tree by randomly selecting attributes as split nodes and assigning random classes to each leaf. It had both numeric and nominal feature types that could be customized.
- **RBF** (Random RBF generator). This data set was derived from the radial basis function generator [61] by generating a fixed number of random centroids. The generator was devised to offer an alternate complex concept type that was not straightforward to approximate with a decision tree model. A drift is introduced by moving the centroids with constant speed.
- **HYPER** (Rotating HYPERplane). The data were generated from a rotating hyperplane in *d*-dimen-sional space [62]. Hyperplanes are useful for simulating time-changing concepts as we can change the orientation and position of the hyperplane in a smooth manner. The instances are labeled as positive or negative based on their coordinates.

The types of drifts vary from one data set to another. It is a gradual drift in the LED and SEA data sets; a sudden drift in AGR; incremental in RBF; and a recurring drift in HYPER. RTG is a stationary (no drifts) data stream.

### 4.2. Framework

The availability of a scientific benchmarking framework in any research domain enriches the experiment and encourages researchers to conduct further studies and experiments in that field. Evaluation is a key challenge associated with stream mining [63]. With regard to concept drift, there is a well-known software environment called massive online analysis (MOA) [64] that is extensively used for evaluation. It is an open-source software for implementing and running stream learning algorithms with various parameters and was the primary tool used in this study.

The experiments were performed on SANAM [65], a supercomputer hosted by the King Abdulaziz City for Science and Technology. SANAM is a computer cluster comprising standard servers connected via a high-speed network. The cluster consists of 210 servers with 3360 processor kernels, 840 graphic chips, and 26,880 GB of main memory. SANAM consists of 300 nodes, each of which possesses the characteristics listed in Table 1.

### 4.3. Results and Discussion

We used the following settings for all the experiments. The number of instances was one million and the number of trees was 10. We report the results for accuracy and execution time. To ensure consistency in the results, the values were averaged over 10 runs. The experiments were executed on a single node of a SANAM supercomputer.

**Figure 3:**
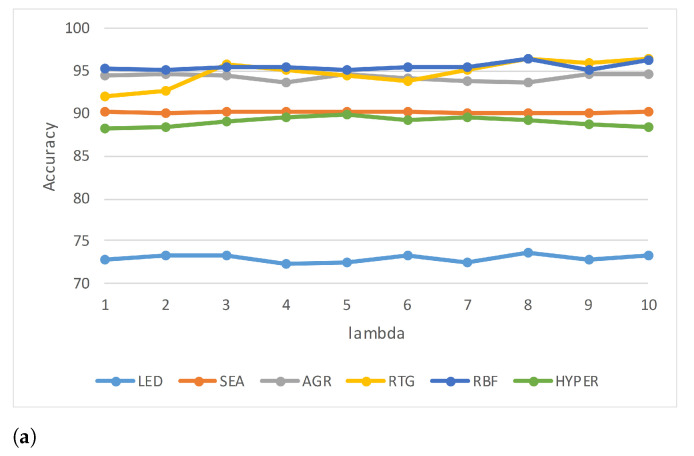

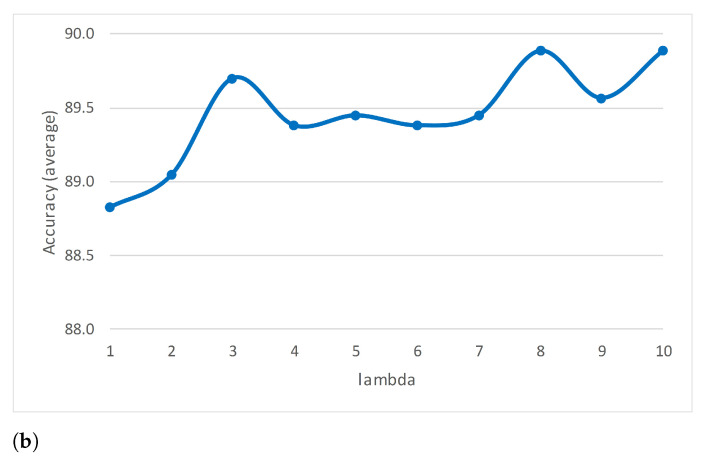
Accuracy for different values of λ of (**a**) each synthetic data set, and (**b**) the overall average.

**Figure 4:**
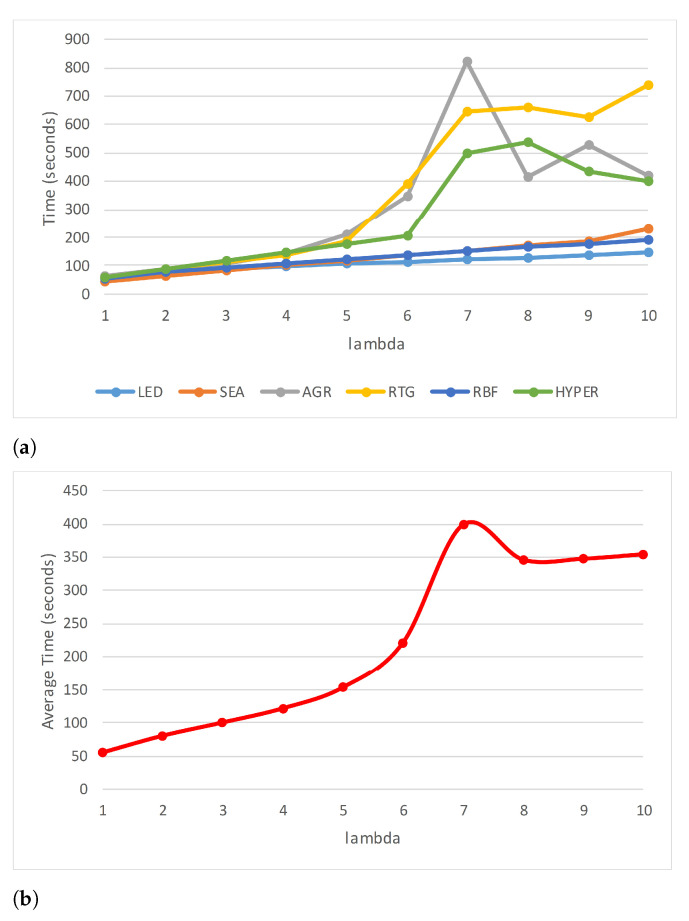
Execution time (in seconds) for different values of λ of (**a**) each synthetic data set, and (**b**) the overall average.

**Figure 5:**
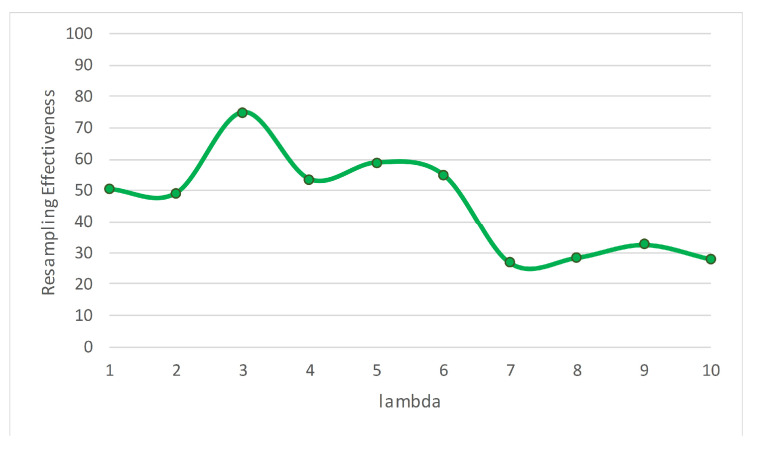
Resampling effectiveness (ρ) for different choices of λ of the synthetic data sets. A higher value of ρ is more desirable.

Table 2 lists the accuracy of each of the data sets for different values of λ. It is seen that the accuracy is restricted to a very narrow range showing very little variation for the different values of λ (see Figure 3a). However, it is interesting to note that the accuracy is not linearly impacted, either positively or negatively, with λ. Figure 3b shows a plot of the average accuracy of the six synthetic data sets. The execution time increases as λ increases (see Table 3), although the AGR and HYPER data sets are exceptions, where the execution time peaks for λ=7 in AGR and 8 in the case of HYPER, and then drops (see Figure 4a). RTG also exhibits a similar behavior; however, the decline is not very drastic. Figure 4b shows the average execution time of all the synthetic data sets. Table 4 lists the values of ρ (Equation (8)) of each of the data sets at different values of λ. According to the values of ρ listed in the table, the best value of λ is either 2 or 3 for five of the six data sets, and for the HYPER data set it is 5. If we average for all the synthetic data sets, the ideal value of λ is 3, and the worst is 7 (see Figure 5).

From Table 2, the best average accuracy is 89.88%, corresponding to λ=8 and 10; however, this accuracy comes at the expense of a large execution time of 346 s, in the case of λ=8 (Table 3). The value of ρ shows that this trade-off of having a slight improvement in the accuracy is not worth the extra execution time.

**Figure 6:**
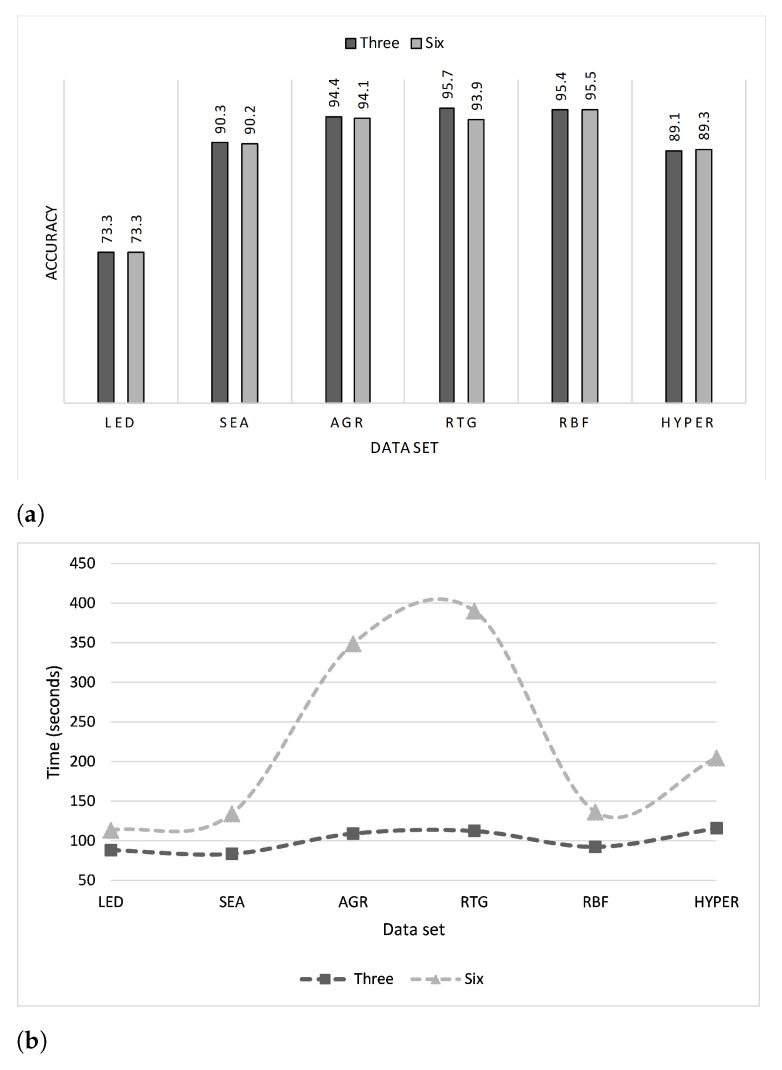
(**a**) Accuracy and (**b**) execution time of each synthetic data set for λ = 3 and 6. The dashed lines are for visual guidance only.

Based on the ρ results (Figure 5), the ARF’s choice of λ=6 as used in [10,53] must be revised. It is clear that λ=3 is a better choice, and there is no need to use a costly method when a less expensive one provides similar accuracy, if not better. Figure 6a,b demonstrate this important outcome.

## 5. Case Study 1

Based on the earlier results, we decided to test the ARF algorithm with our choice of λ on the Amazon customer reviews data set [66] to assess the behavior of the algorithm using a real data set. This data set spans over two decades and contains information from millions of customers and more than 130+ million customer reviews. These reviews express the opinions and experiences of customers on searched and purchased products. The attributes of each review are described in Table 5. Table 6 presents an example of customers’ reviews in the data set.

McAuley et al. [67] extracted several subsets from the Amazon customer reviews data set based on their categories. For our experiment, we decided to choose the data set in the “Books” category (the example presented in Table 6 is for the “Toys” data set). This includes purchased books and customer ID, in addition to customer ratings. More specifications about the selected books data set are shown in Table 7.

After the selection of the data set, the ARF algorithm was executed with values λ=3 and 6. We selected these exact values because the former showed the best ρ in the experiment, whereas the latter is associated with ARF by default (e.g., [10,53]). The accuracy attained in both cases was 79.7%, but with different execution times: with λ=3, the task was completed in 17 min and 45 s whereas it took 28 min and 8 s for λ=6 (under the same conditions). Therefore, by using λ=3, we achieved a speedup of 1.585 times over that when using λ=6.

## 6. Case Study 2

The Hotel Arabic-Reviews Data set (HARD) [68] consists of 93,700 hotel reviews in Arabic that were collected from the Booking.com website covering the period June/July 2016. (The data set is freely available at https://github.com/elnagara/HARD-Arabic-Dataset and was accessed 20 December 2019). The reviews were expressed in Modern Standard Arabic (MSA) as well as dialectal Arabic. For a look at the differences between MSA and dialectal Arabic, see [69]. For sample works on Arabic sentiment analysis, please refer to, e.g., [70,71,72]. Table 8 summarizes some statistics of the data set. Each review has a number of attributes such as hotel no., user type, length of stay, and user rating.

To enhance the process of classification, we added a new derivative feature that indicates the level of feeling associated with each review, either positively or negatively. Each review was broken up into unigram and bigram terms to determine the presence of any of these in a specific sentiment lexicon [73] so that the total number of occurrences reflected the sentiment rate attached to the analyzed review. The ARF algorithm was then applied to the data set using the classification features shown in Table 9.

**Figure 7:**
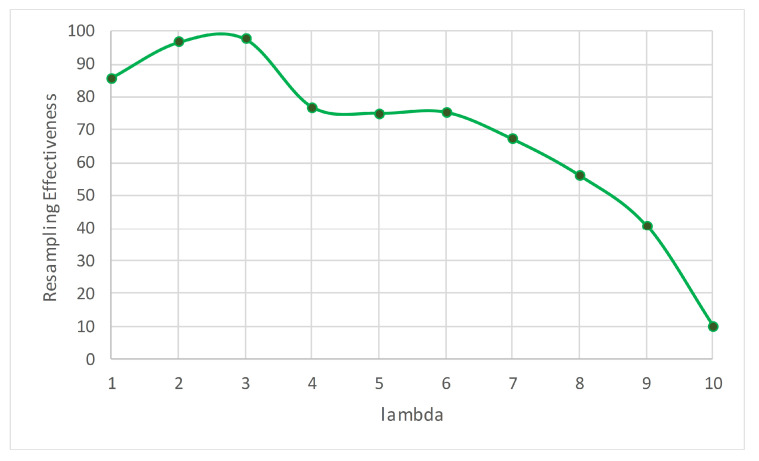
The ρ for different values of λ in the HARD data set.

We executed the ARF algorithm using different values of λ, for comparison. Table 10 lists the classification accuracy, execution time, and ρ for each value of λ. The stream learning time with λ=3 was 10.18 s, and is 22.83 s when λ=6. In other words, the former setting of the algorithm is twice as fast as the latter. From Figure 7, we can see that the best ρ corresponds to λ=3, whereas the second best corresponds to λ=2.

The number of processed records was 105,698 and we achieved our best result in 10.18 s, i.e., approximately 0.01 ms for learning and classifying one review. It ought to be noted that the accuracy was below that reported in [68]; however, this is to be expected as we did not carry out any linguistic preprocessing such as stemming and filtering. In addition, the utilized lexicon is relatively limited as it contains a few thousand words and phrases from the MSA and Saudi dialects. In view of this, the efficacy of stream learning algorithms calls for more attention from researchers in sentiment analysis and emotion recognition.

## 7. Case Study 3

In recent memory, the world has never united as much as it did to combat the Coronavirus disease or COVID-19. This tiny virus caused unprecedented havoc around the world. Many of the world’s economies were paralyzed, and the health systems were on the verge of collapse in other countries. The United States, with one of the most sophisticated health care systems, suffered dearly. This was reflected in US political debates, whether in the traditional or social media. It is not uncommon to see verbal disputes on social media between the president of the United States and the governors of individual states over funding, co-ordination, lack of equipment, and the means to handle the dire situation.

It can be said that the month of April 2020 witnessed one of the darkest periods for the United States population regarding the effects of COVID-19. As more and more of the population started to feel the pinch, the mood shifted, and this was reflected in the tweets. For this case study, we compiled all COVID-19 related tweets covering the entire month of April 2020. As the number of tweets was huge, we confined them to those by President Trump and the governors of the fifty U.S. states. The total number of tweets collected was approximately 10,000. Our objective was to perform an aspect-based sentiment analysis, which is a text analysis technique that breaks down the text into aspects (attributes or components of a service) and then assigns a sentiment level (positive, negative, or neutral) to each. We planned to conduct a real-time analytical study of the compiled tweets to assess the sentiments contained therein.

We used TextBlob (https://textblob.readthedocs.io/en/dev/ accessed on 21 May 2020), which is a Python library, for processing textual data to assess the sentiments of the tweets. This library covers different natural language processing (NLP) tasks such as noun phrase extraction, part-of-speech tagging, and tokenization. Many works, such as [74,75,76], utilized this library for NLP tasks; however, it lacks the ability to analyze the dependency between tokens and extract their syntactic relationships. This is very important in aiding the process of associating feelings with related aspects. For this, another library was used to overcome the shortcomings of TextBlob. SpaCy (https://spacy.io/ accessed on 21 May 2020), also a Python-based library, has dependency-parsing capability and other linguistic features.

**Figure 8:**
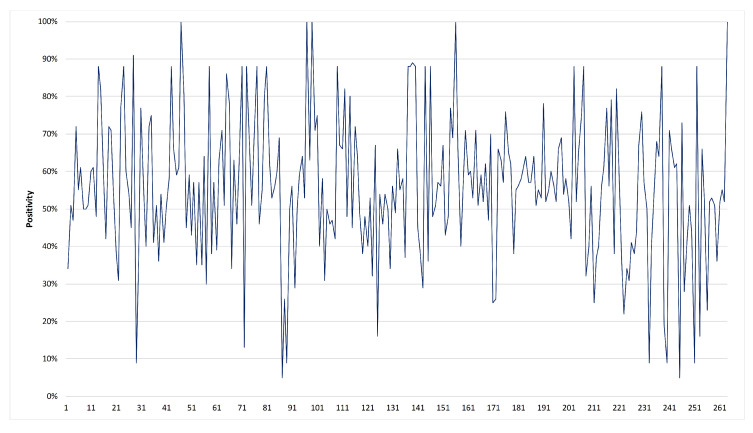
Positivity of 263 tweets of President Trump during April 2020. The tweets are in chronological order.

In sentiment analysis, polarity refers to identifying sentiment orientation (positive, negative, or neutral) in the written/spoken language. A language contains expressions that are objective or subjective. Objective expressions are facts, while subjective expressions are opinions that describe someone’s feelings toward a specific subject or topic. For example, “this apple is red”, is objective; whereas the sentence “this apple is delicious” is subjective. Polarity and subjectivity of the text are important metrics for assessing the sentiments of the mined text (tweets in our case). We define a new measure, positivity, which combines both factors. It is given by Equation (10),

(10) $$\mathrm{positivity}=\left(\frac{{}^{1}\;{/}_{2}\cdot \left(1+\mathrm{polarity}\right)+\mathrm{subjectivity}}{2}\right),$$

where we assume polarity∈{−1,0,1} for respective negative, neutral, and positive sentiments. The subjectivity is 0 if the text is very objective and 1 otherwise. The positivity measure ∈[0,1], can be applied to a paragraph, sentence, or even a clause. More importantly, it can be used for real-time monitoring of a particular aspect and predicting the future sentiment related to it. Therefore, the positivity of the text surrounding some aspects may be used for tagging them. These tagged aspects are then used for forecasting future sentiments by using a classification algorithm such as ARF. Figure 8 shows the positivity of President Trump’s tweets during the month of April 2020.

**Figure 9:**
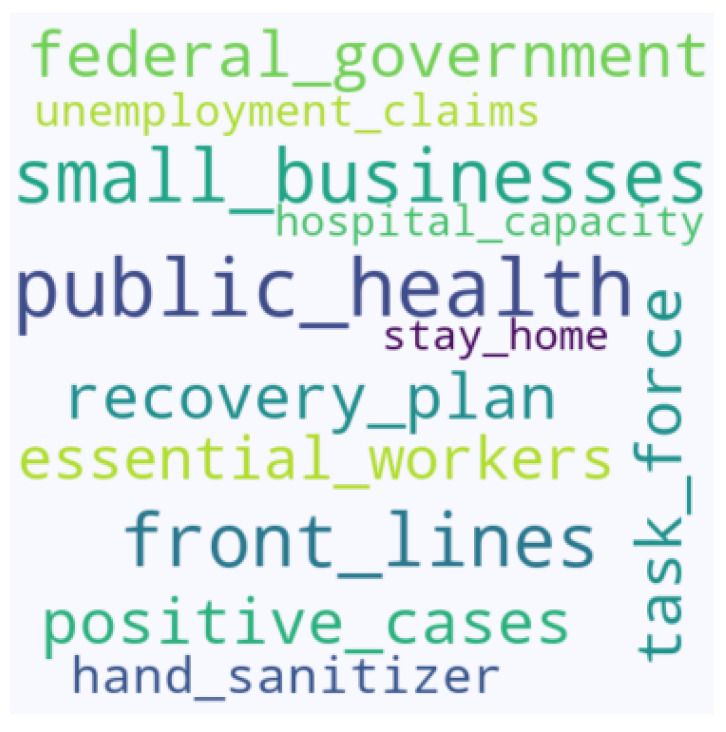
Word cloud for the top most discussed aspects in the compiled COVID-19 tweets. The font size relays the importance of each tag shown.

**Figure 10:**
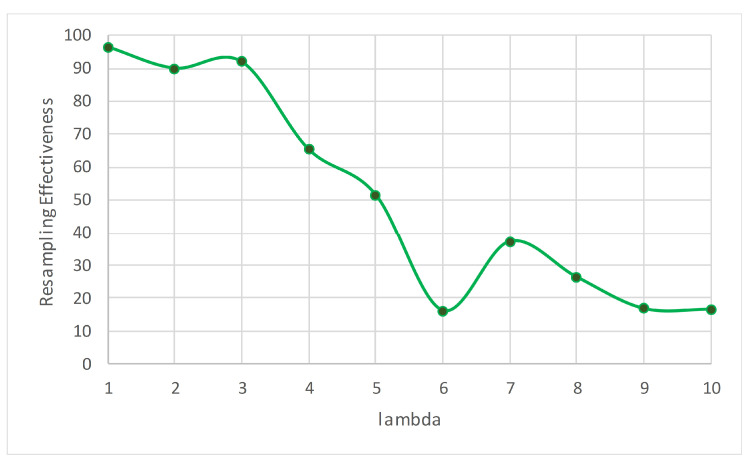
The ρ for different values of λ for the compiled tweets.

We processed the collected tweets by automatically extracting the aspects. Figure 9 lists some of the most-discussed aspects in the compiled COVID-19 tweets. We then continuously examined the evolution of sentiments towards the aspect. For this, we applied the ARF algorithm using different values of λ to predict the future sentiments related to the extracted aspects. Table 11 lists the accuracy, execution time, and ρ for each value of λ when performing the aspect-based sentiment analysis. Figure 10 shows the plot of ρ for different values of λ. Here, our best choice is λ=1, closely followed by λ=3. On the other hand, λ=6 is one of the worst choices. As the execution time was negligible (in ms in Table 11), we note that the best accuracy corresponds to λ=3.

## 8. Conclusions

Big data has become an important topic in the computing world. The breakthrough capabilities of big data analytics have reshaped many aspects and approaches of employing technology in our everyday lives. Thus, concept drift is a critical issue in predictive analysis applications.

The nature of stream learning environments requires effective handling of drifting concepts. Therefore, the purpose of this study was to investigate the issue of concept drift within the domain of random forest to enhance the mechanisms for building an accurate and efficient prediction model based on stream learning. In particular, this research illustrated the importance of resampling effectiveness in such domains.

The adaptive random forest algorithm showed promising results in terms of its accuracy and ability to deal with various types of drift. When the data size is large, as in stream learning, the algorithm can be approximated to a Poisson distribution. This distribution is specified by a single parameter λ, which impacts the resampling process. An issue we tried to address in this work is how to improve the resampling process with replacement. Given that, we proposed a new measure: resampling effectiveness, and denoting it ρ. The objective was to measure the most efficient method by which the system adapts to concept drift. Empirically, we found that using λ=3 was more appropriate for building an efficient adaptive random forest model than λ=6, as suggested in earlier studies. This was confirmed by testing our measure on different data sets used in this work.

We used three case studies with different data sets to confirm our choice of λ. These were: the Amazon customer reviews data set, the Hotel Arabic-Reviews data set, and the COVID-19 related tweets from the United States president and state governors in the United States. For the hotel reviews data set, we chose the field of Arabic sentiment analysis, as we noticed that stream learning was not sufficiently represented in the literature related to the Arabic language. All three studies confirmed our proposed method of enhancement of the random forest algorithm, reflecting its effectiveness in processing three different data sets in two different languages.

In the future, we plan to continue investigating the random forest algorithm and concept drift phenomenon by working on state-of-the-art big data analytics frameworks. Of course, there is a clear research gap in the literature in this area, which includes ARF as well. Furthermore, we plan to investigate other data-related issues, such as dealing with noisy data.

## Figures and Tables

**Table 1:** Specifications of each of the 300 nodes in the SANAM supercomputer.

Characteristic	Description
OS	Linux SUSE Enterprise
CPU	Two Xeon E5-2650 8Cores 2 GHz
GPU	Two AMD FirePro S10000
Memory	128 GB RAM DDR3
Network infrastructure	InfiniBand FDR
Scheduler	Slurm 2.6
File system	Lustre

**Table 2:** Accuracy of each of the synthetic data sets for different values of λ. All values are averaged over 10 runs.

λ	LED	SEA	AGR	RTG	RBF	HYPER	Avg
1	72.8	90.2	94.5	92.0	95.3	88.2	88.83
2	73.3	90.1	94.7	92.6	95.2	88.4	89.05
3	73.3	90.3	94.4	95.7	95.4	89.1	89.70
4	72.2	90.3	93.7	95.2	95.4	89.5	89.38
5	72.4	90.2	94.7	94.4	95.1	89.9	89.45
6	73.3	90.2	94.1	93.9	95.5	89.3	89.38
7	72.4	90.1	93.9	95.2	95.5	89.6	89.45
8	73.5	90.1	93.7	96.4	96.4	89.2	89.88
9	72.7	90.1	94.6	96.0	95.2	88.8	89.57
10	73.3	90.2	94.6	96.5	96.3	88.4	89.88

**Table 3:** Execution time (in seconds) of each of the synthetic data sets for different values of λ. The execution times are averaged over 10 runs.

λ	LED	SEA	AGR	RTG	RBF	HYPER	Avg
1	58.69	43.17	62.83	56.61	54.09	56.11	55.25
2	79.97	64.91	85.07	88.67	75.53	88.11	80.38
3	88.24	83.52	109.14	112.38	92.37	115.74	100.23
4	98.11	100.12	141.81	136.73	107.39	144.44	121.43
5	108.45	117.63	207.82	186.90	122.57	174.11	152.91
6	113.40	134.29	348.92	390.27	136.62	204.64	221.36
7	121.00	151.01	823.39	646.01	150.88	497.11	398.23
8	128.79	168.26	414.41	661.22	163.50	539.76	345.99
9	136.52	182.88	529.53	624.56	176.65	434.01	347.36
10	147.21	227.29	418.47	740.78	188.46	402.27	354.08

**Table 4:** The ρ of each of the synthetic data sets for different values of λ.

λ	LED	SEA	AGR	RTG	RBF	HYPER	Avg
1	68.02	70.97	90.11	18.18	38.51	18.18	50.66
2	82.07	17.99	98.67	35.78	28.26	33.78	49.43
3	77.22	89.06	82.38	88.12	43.53	69.94	75.04
4	17.14	83.83	18.01	81.00	41.62	81.12	53.79
5	32.17	58.99	90.62	68.24	16.88	87.67	59.09
6	58.58	55.23	54.26	51.74	40.91	70.24	55.16
7	28.89	16.51	14.74	34.47	36.39	29.57	26.76
8	44.63	15.91	17.08	33.86	42.17	17.26	28.48
9	28.44	15.20	60.02	39.48	17.40	34.70	32.54
10	17.92	16.92	70.78	18.18	18.06	26.08	27.99

**Table 5:** Amazon customer reviews attributes.

Attribute	Description
market place	country code
customer ID	customer random identifier
review ID	review unique ID
product ID	product identification number
product parent	root product
product title	product’s title description
product category	product’s broad category
star rating	review’s rating value
helpful votes	number of positive votes
total votes	number of total votes
vines	is it a vine review
verified purchase	review by purchasing customer
review headline	review’s title
review body	review’s text
review date	review’s submission date

**Table 6:** Sample of Amazon customer reviews data set. The records are long; therefore, to conserve space, we removed the attributes of “Marketplace”, “Customer ID”, “Review ID”, “Product ID”, and “Product parent”.

Product		Star	Votes			Verified	Review		
Title	Cat.	Rating	Help.	Total	Vine	Purch.	Head.	Body	Date
Monopoly Jr Board Game	Toys	5	0	0	N	Y	Five Stars	Excellent!!!	2015-08-31
Super Jumbo Playing Cards by S&S Worldwide	Toys	2	1	1	N	Y	Two Stars	Cards are not as big as pictured	2015-08-31
Big Bang Cosmic Pegasus (Pegasis) Metal 4D High Performance Generic Battling Top BB-105	Toys	3	2	2	N	Y	Three Stars	To keep together, had to use crazy glue	2015-08-31
Fun Express Insect Finger Puppets 12ct	Toys	5	0	0	N	Y	Five Stars	I was pleased with the product	2015-08-31
Melissa and Doug Water Wow Coloring Book - Vehicles	Toys	5	0	0	N	Y	Five Stars	Great item. Pictures pop thru and add detail as painted. Pictures dry and it can be repainted.	2015-08-31

**Table 7:** Amazon books data set statistics.

Characteristic	Description
Number of instances	14,356,213
Number of customers	5,430,505
Number of items	1,689,201
Start	14 March 2011 12:00:00 AM
End	12 May 2014 11:59:59 PM
First instance timestamp	1300060800
Last instance timestamp	1399939200

**Table 8:** Statistics of the Hotel Arabic-Reviews Data set (HARD).

Characteristic	Size
Number of reviews	373,772
Number of hotels	1858
Avg. reviews per hotel	264
Max reviews per hotel	5793
Min reviews per hotel	3
Number of users	30,889
Avg. reviews per user	15.8
Number of tokens	8,520,886

**Table 9:** Hotel reviews classification features.

Attributes	Description
hotel_id	hotel identification number
user_type	type of the guest(s)
room_type	reserved room type
nights_number	duration of stay
review_length	length of review (in characters)
sentiment_level	degree of feeling
user_rating	user rating class

**Table 10:** Accuracy, time, and ρ for different values of λ for the HARD data set.

λ	Accuracy	Time (s)	ρ
1	53.7%	5.25	85.83
2	55.0%	7.47	96.59
3	55.3%	10.18	97.58
4	53.1%	13.08	76.93
5	53.0%	15.44	75.09
6	53.3%	22.83	75.39
7	52.7%	29.34	67.18
8	52.2%	45.73	56.14
9	51.2%	61.00	40.81
10	49.5%	99.00	10.00

**Table 11:** Accuracy, time, and ρ for different values of λ for the aspect-based sentiment analysis of the compiled tweets.

λ	Accuracy	Time (ms)	ρ
1	58.6%	110	96.51
2	58.3%	160	90.02
3	58.9%	230	92.01
4	57.7%	440	65.45
5	56.8%	510	51.70
6	54.9%	580	16.16
7	56.4%	660	37.09
8	55.7%	720	26.30
9	55.3%	800	16.72
10	56.5%	840	16.43
